# 

**Published:** 1893-06-24

**Authors:** 


**OUR NEW OFFICES**
428, Strand, W.C., will henceforth be the Office of this Journal.

**Notices of Births, Deaths, and Marriages,** are inserted in this column at a charge of 2s. 6d. for an announcement not exceeding four lines.

### NOTICE TO CORRESPONDENTS.

All MS., Letters, Books for Review, and other matters intended for the Editor should be addressed THE EDITOR, THE LODGE, PORCHESTER SQUARE, LONDON, W.

The Editor cannot undertake to return rejected M.S., even when accompanied by stamped directed envelope.

**Notice to Advertisers and Subscribers.**

All Advertisements, Orders for Copies of THE HOSPITAL, and Business Communications should be addressed to The Publisher, *not to the Editor*, THE HOSPITAL, 428, Strand, W.C.

The Cost of Subscription, post free, is as follows:—Per week, 2½d.; per quarter, 2s. 6d.; per half-year, 5s.; per annum, 8s. 6d. Foreign:—Per annum, 10s. 8d.; payable, in all cases, in advance.

**"Burdett's Hospital Annual," 1893.**

Fourth year of publication. 700 pp. Boards, gilt lettering. A most complete guide to British, Colonial, Indian, and American Hospitals, Dispensaries, Nursing and Convalescent Institutions, and Asylums. Price 5s. Published by THE SCIENTIFIC PRESS (Limited), 428, Strand, W.C.

**NOTICE.—The Index for Vol. XIII. (ending March 1893) is on sale at the Office of this Journal, price 2½d., post free.**

### SCRAPS AND GLEANINGS.

AN Anglo-American Hospital has recently been opened in Rio Janeiro which contains 35 beds.

IT is calculated that electricity in its various applications already gives employment to 5,200,000 people.

OVER 5,000 ratepayers in Marylebone have signed a petition in favour of the Public Libraries Act.

MORE than £200 has been raised by means of a local sale of work in aid of the funds of the Hampstead Home Hospital.

MESSRS. BASS AND CO. have contributed 100 guineas towards the Fourth Quinquennial Fund of the London Hospital now being raised.

THE trustees of Smith's (Kensington Estate) Charity have sent a donation of £300 towards the funds of the Metropolitan Hospital, Kingsland Road, N.E.

IN the Middlesex Report it appears that lunacy is on the increase. The County Asylum states it contains 1,097 inmates, of which number 457 are males, and 640 females.

The committee of the Victoria Hospital for Children have lately made an appeal for funds towards the expenses of their convalescent home at Broadstairs. £2,500 is the sum required.

A RUSSIAN physician has discovered that napthalin as a cure for insect bites is most effective. The drug is employed in a saturated solution in vaseline (which is liquid) and then dropped on the affected part.

THE efforts which have been made in the University of Michigan to amalgamate the regular and the homœopathic departments of medicine have not been successful, and for the present no consolidation can be effected.

THE "Annals of Hygiene" report that 5,000 babies die annually in Philadelphia whose deaths are entirely traceable to the diseases which spring from wrong feeding, and the insufficient nourishment afforded by skimmed milk.

IT is asserted on good authority that the area of the newly discovered gold mines in Africa is not less than 11 miles long, and that there is £315,000,000 worth of gold in sight which can be placed on the market in about 10 years' time.

A MEETING of the representatives of the Farnworth, Little Hulton, and Little Lever Local Boards was held at the end of May at Farnworth, when a draft agreement as to a joint committee for the management of a hospital was submitted.

THE annual meeting of the governors and subscribers of the Free Hospital for Sick Children at Nottingham was held on May 18th. The statement of accounts showed a balance of £70 10s. due to the bank in an expenditure of £1,785 19s. 3d.

THE executioner in the United States has been very busy since the foundation of the Government, but the number of murders has been constantly increasing. In 1888 there were 2,184 homicides, in 1889, 3,567, in 1890 4,290, and in 1891, the numbers rose to 5,906.

A COLLECTION, which contributed £10, was made at Christ Church, Herne Bay, in favour of the Cottage Hospital of that district. At the beginning of the year the balance on the accounts of the hospital were now exhausted, the Committee of the Hospital stated.

ON Thursday, May 27th, the Mayor of St. Helen's gave a dinner to the committee and collectors of the Cottage Hospital. This institution is supported by penny a week contributions, and the subscription for this year amount to £829, the balance at the bank being £500.

DR. DUJARDIN, Beaumetz, recommends the following treatment for obesity. A hot morning bath of Eau de Cologne and water followed by dry rubbing and massage. A dose of 15 grammes of iodide of potassium and 250 grammes of water after each meal. Only three meals a-day and no drinking in between.

IT is proposed at Rochester that a ship should be purchased to be fitted up as a floating hospital in view of a probable visitation of cholera. The barque "Melanchton" was offered at £100 for this purpose, and will be purchased subject to the inspection and subsequent approval of the Medical Officer of Health.

A PRAISEWORTHY effort is being made to provide the sum necessary to complete the purchase of the Caxton wing of Morley House Seaside Convalescent Home, St. Margaret's Bay, Dover. The sum of £600 is required, of which £450 has been contributed. Mr. H. Lloyd has given £25 towards the required sum.

THE fifteenth annual meeting of the Home for Convalescent Children at Portsmouth was held the end of May. The meeting took place in the new premises. There are now 12 beds occupied. The Committee report that the year was commenced with a balance of £57. Over 1,230 children have passed through the home since its foundation.

THE New York *Medical Times* reports that the sanitarians and physicians are combating the most extensive outbreak of typhus fever in the city of Mexico, which disease is carrying off about 300 persons a month. It is to be hoped that the alarm at present felt will be turned into a general overhauling of the now existing sanitation in the city.

THE Devon and Exeter Hospital held its annual meeting last month. It appeared that notwithstanding the increase of income during the last year, and the efforts of the staff to reduce the expenditure of the institution, there is still a deficit of £440 in the accounts. It is hoped that the prompt generosity of the public will preclude the possible necessity of closing a ward.

THE alarming fact is reported in a recent American publication on "Prisoners and Paupers" "that with an increase of 24·5 per cent. in population, the number of inmates of our penitentiaries, jails, and reformatories has increased 45·2 per cent." It is to be noted in comparison that here in England criminal convictions fell from 15,033 in 18[illegible]8 to 9,348 in 1889.

A GRAND gala was held at Wigan on May 22nd, in aid of the funds of the Royal Albert Edward Infirmary and Dispensary for Wigan and the district. The entertainment included two matinée concerts, athletic sports, and a display of fireworks in the evening. The annual report of the infirmary is a record of progress, but subscriptions are still urgently needed to pay off a debt of £1,878 on the building fund.

### BOOKS RECEIVED.

THE SCIENTIFIC PRESS

"Hospital Sisters and their Duties." By Eva C. Lückes, Matron of the London Hospital.

SWAN SONNENSCHEIN AND CO.

"The Origin and Growth of the Healing Art." By Edward Berdoe, M.R.C.S,

"An Introduction to Practical Bacteriology." By Dr. W. Migula, translated by M. Campbell, and edited by H. J. Campbell, M D.

HORACE COX.

"Lyrics." By J. A. Goodchild." 5s. 6d.

W. H. EVERETT AND SON.

"Domestic Medicine and Hygiene." By William J. Russell, M.B. Price 2s. 6d.

HENRY FROWDE.

"Helps to the Study of the Bible."

**Periodicals and Pamphlets.**—The Hygienic Review; The Philanthropist; Annals of the American Academy; Our Little Boys; Childs' Companion; Pocahontas; Little Ruby's Curl; The Child's Guardian; Public Opinion (New York); The Cottager and Artisan; The Rural World; The Publishers' Circular; The Dublin Journal of Medical Science; Talk; Excision of Knee Joint; The Medical Pioneer; The Homœopathic World; The Feeble Minded, Epileptic, Deformed, and Crippled; The Clinical Journal; The Monthly Homœopathic Review; The Charity Organisation Review.

### MEDICAL VACANCIES AND APPOINTMENTS.

**APPOINTMENTS.**

S. H. Adams, M.D.Lond., M.R.C.S.Eng., appointed Medical Officer for the Bedford and Kempston Sanitary District of the Bedford Union. T. R. Bailey, M.D., C.M.Edin., reappointed Medical Officer of Health for Bilston. R. de la P. Beresford, M.D.Glasg., L.R.C.P., L.R.C.S.Edin., reappointed Medical Officer of Health to the Oswestry Town Council. W. B. Betenson, L.R.C.P. Lond., M.R.C.S.Eng., appointed Medical Officer for the Fifth District of the Henstead Union. J. St. C. Boyd, M.D., M.Ch. Irel., appointed Resident House-Surgeon to the Union Hospital, Belfast. W. K. Bullmore, M.D.St.And., M.R.C.S.Eng., appointed Medical Officer of Health for the Falmouth Urban Sanitary District. J. Cameron, L.R.C.S.Edin., reappointed Parochial Medical Officer for Kintore. W. F. Cleaver, L.R.C.P. Lond., M.R.C.S.Eng., appointed Medical Officer of Health for the Phillack Urban Sanitary District. J. G. Clendinnen, L.R.C.S.Irel., L.A.H.Dub., reappointed Medical Officer of Health for the Coseley Urban Sanitary District. W. M. Cox, M.R.C.S. Eng., L.R.C.P.Lond., appointed Resident Surgical Officer to the Children's Hospital, Birmingham. J. W. Dryland, M.R.C.S. Eng., L.M., reappointed Medical Officer of Health for Kettering. J. L. Firth, M.D.Lond., L.R.C.P., M.R.C.S., appointed House-Surgeon to the Bristol Royal Infirmary. J. Fulton, M.B., B.Ch. Irel., appointed Dispensary Surgeon to the Union Hospital, Belfast. A. W. Green, L.R.C.P.Lond., M.R.C.S.Eng., appointed Medical Officer for the Workhouse and Casual Wards of the City of London Union. A. A. Hamilton, L.R.C.P., L.R.C.S.Edin., reappointed Medical Officer of Health for Crowle. A. C. Harkness, L.R.C.P., L.R.C.S.Edin., L.F.P.S.Glasg., appointed Medical Officer for the Whitmore District of the Newcastle-under-Lyme Union. G. Hartridge, F.R.C.S., appointed Lecturer in Ophthalmic Surgery to the Westminster Hospital Medical School. A. B. Hill, M.D.Gressen, L.R.C.P., L.R.C.S.Edin., appointed Medical Officer of Health for the Aston Urban Sanitary District. T. H. Hitchins, M.R.C.S.Eng., L.S.A., reappointed Medical Officer of Health for the Shipston-on-Stour Union. S. Honeywill, L.R.C.P.Edin., M.R.C.S.Eng., appointed Medical Officer of the Workhouse of the Cosford Union. H. Horsfall, M.D.St.And., M.R.C.S.Eng., reappointed Medical Officer of Health for the Bedale Rural Sanitary District. T. W. Kyle, M.D.Irel., M.Ch., appointed Medical Officer of Health for Ashby-de-la-Zouch. D. C. McArthur, M.R.C.S.Eng., L.R.C.P. Lond., appointed Medical Officer for the Sixth District of the South Molton Union. J. McLiesh, M.B., B.Ch.Irel., appointed *pro tem.* House-Surgeon to the Union Hospital, Belfast. W. J. Maurice, M.B., B.Ch.Oxon., L.R.C.P.Lond., appointed Surgeon to the Royal Berks Hospital, Reading. A. M. Palmer, L.R.C.P.Edin., M.R.C.S.Eng., appointed Medical Officer of Health to the Whittington Local Board. A. A. Philip, M.B., C.M.Aberd., appointed Medical Officer and Public Vaccinator for the Dent District of the Sedbergh Union. Mr. C. Read, appointed Medical Officer for the No. 1 District of the City of London Union. G. E. Rennie, B.A.Syd., M.D.Lond., M.R.C.S.Eng., Pathologist to Prince Alfred Hospital, Sydney, appointed Assistant Physician to Sydney Hospital, New South Wales. E. S. Robertson, L.R.C.P.Lond., M.R.C.S., appointed Medical Officer of Health to the Stourport Local Board. R. Settle, M.D.Glas., L.R.C.S.Edin., reappointed Medical Officer

### MEDICAL APPOINTMENTS.

### QUEEN CHARLOTTE'S LYING-IN HOSPITAL,
Marylebone Road, N.W.

RESIDENT MEDICAL OFFICER REQUIRED.

### ROYAL VICTORIA HOSPITAL, Bournemouth.

### THE GENERAL HOSPITAL, BIRMINGHAM.

### COUNTY ASYLUM, LANCASTER.

WANTED, an ASSISTANT MEDICAL OFFICER who shall act as Pathologist. Applicants must be duly qualified and unmarried. Salary £100, increasing by annual instalments of £25 to £200 per annum, with Furnished Apartments, Board, &c.

Address, with testimonials, not later than June 24th, Dr. CASSIDY, Medical Superintendent. (1,328)

### THE HOSPITAL FOR SICK CHILDREN,

MEDICAL VACANCIES AND APPOINTMENTS—(*continued*)

of Health for the Astley Bridge Local Board. S. A. Shiach, M.B.Edin., appointed Dispensary Surgeon to the Bradford Infirmary. R. W. Soper, M.R.C.S.Eng., L.S.A., reappointed Medical Officer of Health for the Dartmouth Borough and Port. T. Smailes, M.D.St.And., L.R.C.P.Edin., M.R.C.S.Eng., reappointed Medical Officer of Health for South Crosland. G. M. Swinhoe, L.R.C.P.Lond., M.R.C.S.Eng., reappointed Medical Officer of Health to the New Swindon Local Board. H. H. Tipping, M.R.C.S.Eng., L.R.C.P.Lond., appointed Resident Medical Officer to the Children's Hospital, Birmingham. C. H. Whiteford, M.R.C.S.Eng., L.R.C.P.Lond., appointed House-Surgeon to the Coventry and Warwickshire Hospital, Coventry. W. E. Williams, F.R.C.S.Edin., reappointed Medical Officer of Health for the Abertillery Urban Sanitary District. M. C. Wright, L.R.C.P., L.R.C.S.Edin., L.F.P.S.Glasg., appointed Assistant Colonial Surgeon to the Gold Coast Colony, West Africa. Sibley, W. Read, has been appointed Assistant Dental Surgeon to the National Dental Hospital.

### VACANCIES.

The following vacancies are announced this week, the amount of salary in each case being given after the name of the institution:

**Resident Medical Officers.**

County Asylum, Lancaster. Assistant Medical Officer; unmarried. Salary £100 per annum, increasing by annual instalments of £25 to £200 per annum, with furnished apartments, board, &c. Applications to Dr. Cassidy, Medical Superintendent, by June 24th.

Croydon General Hospital. House-Surgeon; doubly qualified. Salary £100 per annum, increasing £10 yearly to £120, with board and residence. Applications to the Secretary by June 29th.

East London Hospital for Children, Glamis Road, Shadwell, E. House-Surgeon. Board and lodging provided. Applications to the Secretary by July 6th.

General Hospital, Birmingham. Assistant House-Surgeon. No salary; residence, board, and washing. Applications, with registration certificate, to the House Governor by July 1st.

Monkwearmouth and Southwick Hospital, 5, Bedford Terrace, Sunderland. House-Surgeon; doubly qualified. Salary £80 per annum, with board, lodging, &c. Applications to Scott Gunn, Honorary Secretary, by June 30th.

Newport and Monmouthshire Infirmary and Dispensary, Newport, Mon. House-Surgeon; doubly qualified. Salary £100 per annum, with board and residence. Applications to John R. Stone, Secretary, by June 24th.

Liverpool Dispensaries. Assistant Surgeon; unmarried. Salary £80 per annum, with apartments, board, and attendance. Applications to R. R. Greene, Secretary, by July 24th.

Queen Charlotte's Lying-in Hospital, Marylebone Road, N.W. Resident Medical Officer. Appointment for four months. Salary at the rate of £60 per annum, with board and residence; and Physician to Out-patients. Applications to G. Owen Ryan, Secretary, by June 24th.

**Non-Resident Medical Officers.**

City of London Hospital for Diseases of the Chest, Victoria Park, E. Assistant Physician; must be Fellows or Members of the Royal College of Physicians of London. Applications to the Secretary, at the office, 24, Finsbury Circus, E.C.

Royal Victoria Hospital, Bournemouth. Ophthalmic Surgeon. Applications to the Secretary by July 1st; and a House-Surgeon-Secretary. Salary £100 per annum and board for the latter. Applications to the Chairman of Committee by July 1st.

St. Bartholomew's Hospital and College. Demonstrator of Chemistry and Assistant Demonstrator of Chemistry. Applications to Dr. T. W. Shore, Warden, by June 30th.

University of London, Burlington Gardens, W. Examiner in Surgery. Salary £200 per annum. Applications to the Registrar by June 24th.

Westminster Hospital, Broad Sanctuary, S.W. Assistant Physician. Applications to House Committee before June 27th.

### PASS LIST.—University of London.

M.B. PASS EXAMINATION: MAY, 1893.

FIRST DIVISION.—G. F. Bergin, Bristol Medical School; F. P. S. Cresswell, B.Sc., Guy's Hospital; W. Edgecombe, University Colleges—Liverpool and London; H. Langdale, Owens College and Manchester Royal Infirmary; A. J. Martin, Mason College and General Hospital, Birmingham; A. Paine, St. Mary's Hospital; C. Paine, St. Mary's Hospital; N. C. Ridley, St. Mary's Hospital; and C. H. Roberts, St. Bartholomew's Hospital.

SECOND DIVISION.—C. B. Braithwaite, Guy's Hospital; J. B. Byles, University College; R. H. Castellote, University College; I. Costa, University College; A. J. Edge, St. Bartholomew's Hospital; C. A. Fuller, St. Mary's Hospital; R. C. Gully, St. Bartholomew's Hospital; E. G. Hall, Medical School and Royal Infirmary, Bristol, and Guy's Hospital; F. Hazell, Guy's Hospital; F. Johnson, St. Bartholomew's Hospital; H. D. Levick, St. Thomas's Hospital; A. G. Levy, University College; J. H. Muncaster, Guy's Hospital; J. E. Ramsay, University College; J. L. Sawers, University College; R. M. Smyth, St. Mary's Hospital; J. H. Sproat, Queen's and Mason Colleges, Birmingham; G. R. F. Stilwell, St. Thomas's Hospital; A. C. Ta'Bois, St. Bartholomew's Hospital; G. A. Watson, University College; S. Williams, University College; and J. Williamson, St. Bartholomew's Hospital.

